# Simple, Low-Cost Fabrication of Highly Uniform and Reproducible SERS Substrates Composed of Ag–Pt Nanoparticles

**DOI:** 10.3390/nano8050331

**Published:** 2018-05-15

**Authors:** Tao Wang, Juhong Zhou, Yan Wang

**Affiliations:** Provincial Key Laboratory of Functional Coordination Compounds and Nanomaterials, School of Chemistry and Chemical Engineering, Anqing Normal University, Anqing 246001, China; wangtao@aqnu.edu.cn (T.W.); wangyan@aqnu.edu.cn (Y.W.)

**Keywords:** germanium, surface-enhanced Raman scattering, Ag–Pt nanoparticles

## Abstract

Ag–Pt nanoparticles, grafted on Ge wafer, were synthesized by the galvanic replacement reaction based on their different potentials. Detailed characterization through scanning electron microscopy (SEM), energy-dispersive X-ray spectrometry (EDS) and X-ray photo-elelctron spectroscopy (XPS) proved that Ag–Pt nanoparticles are composed of large Ag nanoparticles and many small Pt nanoparticles instead of an Ag–Pt alloy. When applied as surface-enhanced Raman scattering (SERS) substrates to detect Rhodamine 6G (1 × 10^−8^ M) or Crystal violet (1 × 10^−7^ M) aqueous solution in the line mapping mode, all of the obtained relative standard deviation (RSD) values of the major characteristic peak intensities, calculated from the SERS spectra of 100 serial spots, were less than 10%. The fabrication process of the SERS substrate has excellent uniformity and reproducibility and is simple, low-cost and time-saving, which will benefit studies on the platinum-catalyzed reaction mechanisms in situ and widen the practical application of SERS.

## 1. Introduction

Due to the outstanding ability of surface-enhanced Raman scattering (SERS) to offer unique vibrational signatures of Raman-active analyzed molecules [1,2], it has been regarded as a perfect, powerful, analytical technology and applied to a wide range of fields, including chemical sensing [3], environmental monitoring [4], diagnostics [5,6], biodetection [7,8], forensic science [9], homeland security and defense [10]. Although many SERS substrates with excellent enhancement effects have been prepared, the practical applications of SERS are still limited. The main reasons for this result are the complex fabrication process of SERS substrates and the poor signal reproducibility. Only SERS substrates with good stability can produce reproducible SERS signals. The reliability and accuracy of the information obtained from SERS can also been ensured. 

The composition of SERS-active materials is the key factor that determines the SERS performance of a substrate. Noble metals, such as silver and gold, are widely applied in SERS substrates due to their localized surface plasmon resonance, generally lying in the visible optical range, where the most frequently used lasers are. In particular, SERS substrates composed of Ag nanostructures are recognized as possessing outstanding SERS performance [11]. However, when the Ag nanoparticles are in contact with oxidants, thiols [12], halide ions [13], acid [14] or treated by UV irradiation [15] or heating [16], they are oxidized, resulting in morphological change and performance depreciation [17]. In order to improve the stability of SERS substrates based on Ag nanoparticles, a metal or oxide with better stability was used to cover or alloy with silver. It is well known that Pt not only has excellent surface stability and good biocompatibility [18], but it also has wide application as a catalyst and electrode in surface science and electrochemistry; hence, it has been utilized to cover Ag to obtain better performances [19,20].

Various techniques have been adopted to fabricate the SERS-active substrates with high-performance, including electron beam lithography [21], self-assembly [22], focused ion beam [23], nanosphere lithography [24], nanoimprinting [25], block copolymer template [26], and oblique angle deposition [27], but disadvantages in these fabrication methods hamper the practical application of SERS: (1) the fabrication procedures are time-consuming and complex; (2) the surfactants or polymers used in the preparation of substrate unavoidably deteriorate the SERS activity; (3) professional training in sample preparation and equipment operation are absolutely necessary in some fabrication methods, which may put SERS beyond the reach of all but research labs; (4) expensive, specialized instruments are required in some fabrication process, which makes these substrates high-cost and restricts their popularity.

Hence, it is highly desirable to synthesize the highly uniform and reproducible SERS substrates using a simple, low-cost and time-saving method. Galvanic replacement, also named galvanic displacement, is a electrochemical reaction in which a metal ion in solution displaces atoms from a solid metal or semiconductor surface [28]. Without an external voltage source and reducing agents, the galvanic replacement reaction can occur spontaneously [29]. Due to the simplicity of operation, cost-effectiveness and lack of elaborate equipment, the galvanic replacement reaction has been widely used in the fabrication of SERS substrates [30,31].

In this paper, we successfully fabricated a SERS substrate composed of Ag–Pt nanoparticles (NPs) with the galvanic replacement reaction. The fabrication method is simple, low-cost and time-saving. The reagents and equipment used are easily obtainable. In the SERS detection of dilute Rhodamine 6G (R6G) or Crystal violet (CV) aqueous solution with the line mapping mode, the as-prepared SERS substrates not only indicated excellent SERS activity, but also showed excellent uniformity and reproducibility. All the relative standard deviation (RSD) values of the major characteristic peak intensities were less than 10%.

## 2. Materials and Methods

### 2.1. Materials

Analytical grade AgNO_3_ and H_2_PtCl_6_ were purchased from Sinopharm Chemical Reagent Co., Ltd. (Beijing, China). Highly doped n-type Ge wafers with dopant (Sb) at a concentration of about 1 × 10^18^ cm^−3^ were purchased from Hefei Kejing Materials Technology Co., Ltd. (Hefei, China).

### 2.2. Fabrication of SERS Substrate

The Ge wafer was cut into square pieces of 1 × 1 cm^2^, rinsed with pure water and then desiccated with gentle N_2_ flow. The freshly cleaned wafer was reacted with 20 mL 1 mM AgNO_3_ aqueous solution for 10 min and then taken out. After being rinsed with pure water and absolute ethanol, the Ge wafer was immersed into 20 mL 0.2 mM H_2_PtCl_6_ solution for 5 min and then taken out. Finally, the wafer grafted with Ag–Pt NPs was rinsed with pure water and dried by a gentle N_2_ flow.

## 3. Results and Discussion

### 3.1. Characterization Ge Wafer Grafted with Ag–Pt NPs

**Figure 1:**
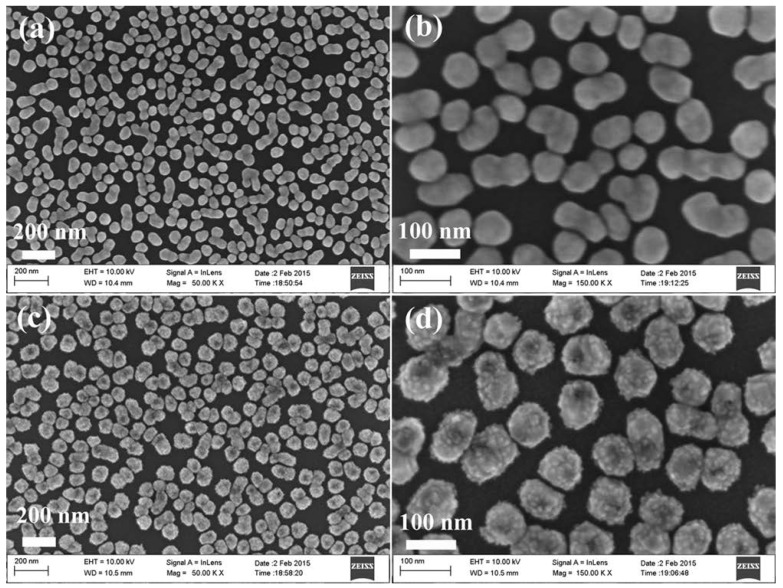
(**a**) Low magnification and (**b**) high magnification SEM images of Ag nanoparticles grafted on the Ge wafer; (**c**) Low magnification and (**d**) high magnification SEM images of Ag–Pt nanoparticles grafted on the Ge wafer.

Figure 1 shows the morphologies of the particles grown on the Ge wafer at different steps using a scanning electron microscope (SEM). The low and high magnification image of the product obtained after the Ge wafer reaction with AgNO_3_ solution is shown in Figure 1a,b, and the Ge wafer grafted with many Ag NPs is revealed. The average diameter of the Ag NPs was about 90 nm. After the reaction with H_2_PtCl_6_ solution, the Ge wafer was grafted with the Ag–Pt NPs. Figure 1c,d show low and high magnification images. It can be observed in Figure 1d and Figure S1 that there were many small nanoparticles with an average diameter of about 8 nm on the surface of the Ag–Pt nanoparticles. Through the energy-dispersive X-ray spectrometry (EDS) analysis of the Ag–Pt NPs grafted onto the Ge wafer (Figure S2), it was shown that the Pt content in the Ag–Pt NPs was about 8.57%.

**Figure 2:**
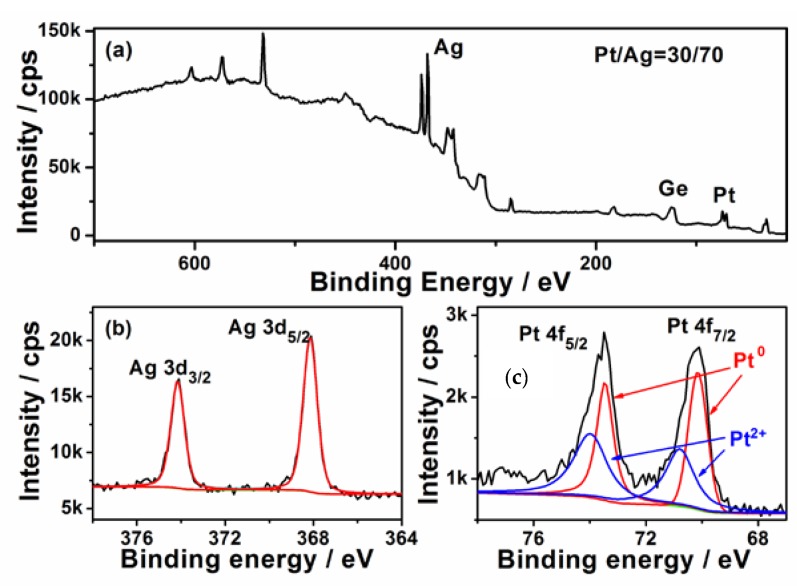
(**a**) XPS survey spectrum of Ag–Pt NPs/Ge; (**b**,**c**) the high solution XPS spectra of Ag 3d and Pt 4f.

The surface composition of the Ge wafer grafted with Ag–Pt nanoparticles was analyzed with X-ray photo-elelctron spectroscopy (XPS). Figure 2a shows its survey XPS spectrum, which reveals the Ag and Pt in the Ge wafer. The high solution XPS spectrum of Ag 3d is shown in Figure 2b; the two peaks at 367.6 and 373.6 eV are attributed to the binding energies of Ag 3d_5/2_ and Ag 3d_3/2_, which is ~0.7 eV less than the corresponding values of bulk metal [32] and the Ag NPs grafted on Ge wafer through the galvanic replacement reaction [33]. This phenomenon has been also observed in Ag–Pt/SiO_2_ due to the transfer of electrons from Pt to Ag [34]. According to Figure 2c, the XPS spectrum of Pt 4f is composed of two couple peaks; the couple peaks at about 70.2 and 73.5 eV are assigned to Pt^0^, while the couple peaks at about 70.8 and 74.0 eV are attributed to Pt^2+^. The existence of Pt^2+^ can be attributed to the incomplete reduction of ${\mathrm{PtCl}}_{6}^{2-}$ [35]. Through the XPS analysis, the content of the Pt in the surface of Ag–Pt nanoparticles was shown to be about 30% which is much higher than the value obtained from the EDS spectrum analysis, confirming that the surface of Ag–Pt NPs was enriched with Pt [36]. The difference in the element composition between XPS (a surface analysis tool with a sampling depth of about 30 Å) and EDS (a bulk analysis technique) measurement is strong evidence that these small nanoparticles in the Ag–Pt nanoparticle were only composed of Pt.

**Figure 3:**
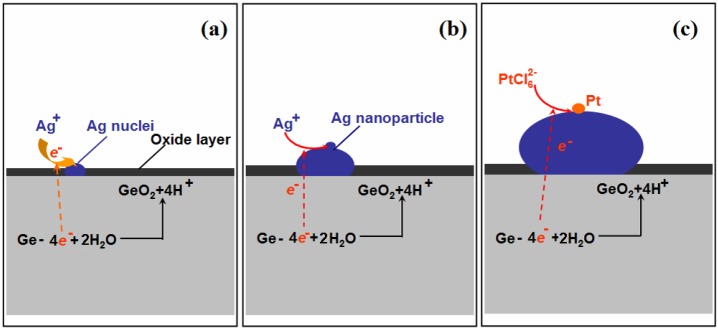
Schematic illustration of the growth process of Ag–Pt NPs on the Ge wafer. (**a**) the formation of Ag nuclei; (**b**) the growth of Ag nanoparticle and (**c**) the formation of Pt nanoparticle on the surface of Ag nanoparticle.

Figure 3 shows the formation of Ag–Pt NPs on the Ge wafer; when the Ge wafer was immersed in the AgNO_3_ solution, the silver ions were immediately reduced by the surface electrons of Ge, resulting into the formation of Ag nuclei (Figure 3a). Although the surface electrons of the Ge wafer were consumed, because the potential of the valence band of Ge (−0.5 V versus a standard hydrogen electrode (SHE)) is smaller than the potential of the Ag^+^/Ag couple (>0.385 V versus SHE for AgNO_3_ solutions with a concentration larger than 1 × 10^−4^ mM), the electrons of Ge were able to reduce silver ions continuously and the Ag nuclei became larger (Figure 3b). Meanwhile, the Ge were oxidized into GeO_2_ through the loss of electrons [33]. The result of this replacement reaction was that the Ge wafer was grafted by Ag NPs.

Because the potential of the valence band of Ge is also smaller than that of ${\mathrm{PtCl}}_{6}^{2-}$/Pt couple (>0.614 V for ${\mathrm{PtCl}}_{6}^{2-}$ solution with a concentration larger than 1 × 10^−4^ mM), the ${\mathrm{PtCl}}_{6}^{2-}$ anions were reduced by the electrons of Ge to form Pt NPs (Figure 3c). It should be mentioned that the potential of the Ag^+^/Ag couple (<0.5636 V for the concentration of Ag^+^ lower than 1 × 10^−4^ M) is also lower than that of ${\mathrm{PtCl}}_{6}^{2-}$/Pt couple, which means that ${\mathrm{PtCl}}_{6}^{2-}$ anions should also be reduced by Ag. However, the potential difference between the ${\mathrm{PtCl}}_{6}^{2-}$/Pt couple and Ag^+^/Ag couple was small, which suggests that the reaction rate between ${\mathrm{PtCl}}_{6}^{2-}$ and Ag is slow. Furthermore, due to Ag being an excellent conductor, the electrons of Ge with much stronger reductive power can transmit through Ag nanoparticles quickly and reduce the ${\mathrm{PtCl}}_{6}^{2-}$ readily to form the Pt nanoparticles. Consequently, ${\mathrm{PtCl}}_{6}^{2-}$ anions were reduced by the electrons from the Ge and formed Pt nanoparticles on the surface of Ag nanoparticles.

Because the Pt can barely undergo solid–solid diffusion with Ag when the temperature is below 900 K [37], the Ag–Pt nanoparticle grafted on the Ge wafer through the galvanic replacement reaction was composed of large Ag nanoparticles and many small Pt nanoparticles instead of being a Ag–Pt alloy, which has been well demonstrated by the above results of SEM, EDS and XPS.

### 3.2. SERS Activity and Uniformity

To assess the SERS activity and uniformity of the as-prepared substrates, the R6G with well-established vibrational spectroscopy was first selected as the analyzed molecule to collect the SERS spectra.

**Figure 4:**
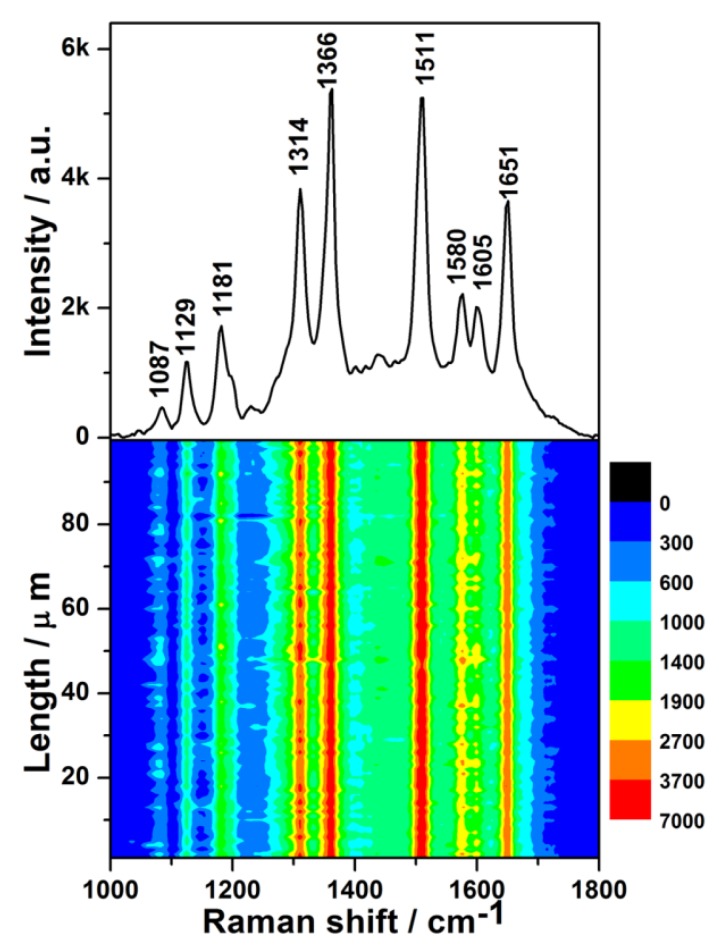
(**Upper**): surface-enhanced Raman scattering (SERS) spectrum of 1 × 10^−8^ M Rhodamine 6G (R6G) solution on the Ge wafer grafted with Ag–Pt nanoparticles. (**Lower**): SERS contour from line mapping of 100 spots.

The SERS spectrum of dilute R6G aqueous solution with a concentration of 1 × 10^−8^ M is shown in the upper half of Figure 4, which is obtained from one random spot of the as-prepared SERS substrate. Not only is the SERS spectrum of the R6G solution similar to its normal Raman spectrum (Figure S3), but also, the barely-observed peaks at 1087 and 1605 cm^−1^ in normal Raman spectrum are prominent. It has been fully demonstrated that the substrate possesses remarkable SERS activity.

The lower half of Figure 4 is the contour of the SERS spectra for 1 × 10^−8^ M R6G, which was plotted from 100 SERS spectra measured by the Raman-line mapping mode at 1 μm intervals. In all of the 100 SERS spectra, the intensities of the same characteristic peak were similar, which demonstrates that all spots of the SERS substrate in the scanned area exhibited an excellent enhancement effect.

To further evaluate the uniformity of the measured SERS signals semi-quantitatively, the relative standard deviation (RSD) values of the characteristic Raman peaks were calculated. The RSD values of the peaks at 1314, 1366, 1511, and 1651 cm^−1^ (Figure S4) were found to be 9.09%, 8.37%, 9.28%, and 8.99%, respectively. All of the obtained RSD values were less than 10%, demonstrating excellent uniformity [38]. It was found that the distributions of the intensities of the major characteristic peaks were log-normal (Figure S5), which indicated that many hotspots existed in the scanned area of the SERS substrate [39]. In addition, the narrow distribution of peak intensities further manifested the uniformity of SERS signals. To evaluate the enhancement activity of the as-prepared SERS substrate quantitatively, the average enhancement factor (EF) was calculated for the characteristic peak at 1511 cm^−1^ according to the following equation [40]:

$$\mathrm{EF}=\frac{I_{\mathrm{SERS}}N_{0}}{I_{0}N_{\mathrm{SERS}}},$$

where *N*_0_ and *N*_SERS_ are the numbers of R6G molecules in the normal Raman measurement and in the SERS measurement, respectively; and *I*_0_ and *I*_SERS_ are the peak intensity in the normal Raman measurement and the average peak intensity in the SERS measurement, respectively. The calculated EF was 9.1 × 10^6^ (the detailed calculation process is shown in supporting information).

The excellent enhancement effect of the as-prepared SERS substrate may have resulted from the combined action of the following factors. Firstly, because the SERS intensity decreased with an increase in Pt content in the Ag–Pt nanoparticle [20], the low content of Pt (8.57%) guaranteed that the prepared SERS substrate still exhibited a high enhancement effect. Secondly, because the SERS effect showed strong distance-dependence [41], the clean and increased surface Ag–Pt NPs, derived from the formation of many Pt nanoparticles on an Ag nanoparticle, ensured more target molecules were close enough to the surface of the SERS substrate and produced a high enhancement effect. Meanwhile, the surface of Ge wafer was flat, which favoured the production of uniform SERS signals. Finally, the existence of “hot spots” was conducive to an excellent SERS enhancement effect of the substrate. Some distances between Ag–Pt nanoparticles were within approximately 2 nm (Figure 1d and Figure S1), which suggests that the prepared SERS substrate contained some “hot spots” [42].

**Figure 5:**
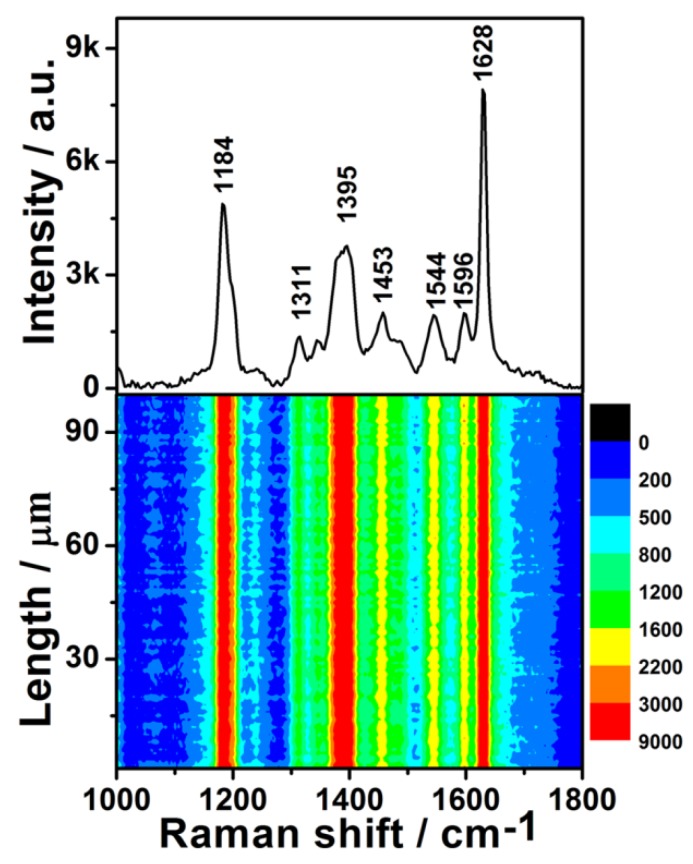
Upper: the SERS spectrum of 1 × 10^−7^ M Crystal violet (CV) solution on the Ge wafer grafted with Ag–Pt nanoparticles. Lower: the SERS contour from line mapping of 100 spots.

To further evaluate the generality of the SERS performance of the as-prepared substrate, the dilute CV aqueous solution was also detected. The upper half of Figure 5 shows the SERS spectrum of 1 × 10^−7^ M CV, which is entirely consistent with previous literature [27]. The SERS contour of the CV aqueous solution is shown in the lower half of Figure 5. All 100 spots demonstrated an excellent enhancement effect. The RSD values of the peak intensities at 1184, 1395, 1544, and 1628 cm^−1^ were 8.34%, 9.25%, 9.30%, and 8.10% (Figure S6), respectively. These values are very close to those of R6G, demonstrating that the preparation of these uniform SERS substrates is reproducible. The intensities of these four characteristic peaks also exhibited a log-normal distribution (Figure S7), further confirming there were hot spots in the scanned area of SERS substrate. Moreover, the distribution width of the peak intensities of CV was almost identical to that of R6G, which further indicated that the fabrication reproducibility is good. Although the enhancement effect of the as-prepared SERS substrates was not as good as the Ag/Ge substrate [33], it showed better stability than the Ag/Ge substrate as well as many other SERS substrates based on colloidal silver. The enhancement effect of the as-prepared substrate did not degrade obviously (Figure S8). 

It is worth pointing out that all SERS signals were collected from the aqueous solution, to acquire accurate information about the molecular structure in solution. It’s good for the study on the solution reaction mechanism through in situ SERS monitoring [43,44]. Because no surfactants or ligands were used in the preparation, the clean surface of Ag–Pt nanoparticles is beneficial to the adsorption of target species for applications in plasmonic-enhanced physical or chemical processes [45].

## 4. Conclusions

An excellent SERS substrate was synthesized successfully by a simple, low-cost and time-saving method, composed of Ag–Pt nanoparticles grafted on Ge wafer. The equipment and reagents used in the fabrication process are readily available. The results of the SERS measurements for dilute R6G and CV aqueous solution fully demonstrated that the as-prepared substrates possessed an excellent enhancement effect. In the SERS detection of R6G and CV aqueous solution with line mapping mode, all the calculated RSD values were smaller than 10%, demonstrating the excellent uniformity and good fabrication reproducibility of the as-prepared SERS substrate. This simple SERS substrate has excellent performance and is a promising contribution to the mechanisms of platinum-catalyzed reactions through in situ SERS monitoring and the practical applications of SERS.

## Supplementary Materials

The following are available online at http://www.mdpi.com/2079-4991/8/5/331/s1, Characterization and SERS Measurement. Figure S1: The high magnification SEM images of Ag–Pt nanoparticles grafted on the Ge wafer. Figure S2: The EDS spectra of the Ge wafer grown with Ag–Pt nanoparticles. Figure S3: The normal Raman spectrum of 0.01 M R6G methanol solution. Figure S4: The intensities of the major peaks in the 100 SERS spectra of R6G solution (1 × 10^−8^ M). Figure S5: Histograms of normalized Raman intensities of R6G (1 × 10^−8^ M) on Ag–Pt NPs/Ge SERS substrate. Figure S6: The intensities of the major peaks in the 100 SERS spectra of 1 × 10^−7^ M CV solution. Figure S7: Histograms of normalized Raman intensities of CV (1 × 10^−7^ M) on Ag–Pt NPs/Ge SERS substrate. Figure S8: The SERS spectrum of 1 × 10^−7^ M R6G solution on Ag–Pt NPs/Ge SERS substrate after a one-year storage in atmospheres at room temperature. The SERS contour from line mapping of 100 spots.
