# Supplementary material for: Simple, Low-Cost Fabrication of Highly Uniform and Reproducible SERS Substrates Composed of Ag–Pt Nanoparticles
Source: Nanomaterials (Basel). 2018 May 15;8(5):331. doi: 10.3390/nano8050331 (PMC5977345; doi:10.3390/nano8050331)
Supplement: Supplementary file 1 [file nanomaterials-08-00331-s001.pdf]

Supporting Information

Simple, low-cost fabrication of highly uniform and reproducible SERS substrates composed of Ag-Pt nanoparticles

Tao Wang, Juhong Zhou* and Yan Wang

Provincial Key Laboratory of Functional Coordination Compounds and Nanomaterials, School of Chemistry and Chemical Engineering, Anqing Normal University, Anqing, 246001, P. R. China

1. Characterization

The morphologies of the samples were performed on a Zeiss Supra 55 field scanning electron microscopy (SEM) operating in high vacuum mode at 10 kV accelerating voltages. The Energy-dispersive X-ray spectrometry (EDS) analysis was carried out on a FEI-quanta 200F SEM with acceleration voltage of 30 kV. The surface state of the as-prepared SERS substrate was studied by XPS measurement (Kratos AXIS UltraDLD ultrahigh vacuum surface analysis system) with Al Kα radiation (1486 eV) as probe. The obtained final spectra were calibrated to the adventitious carbon C 1s peak at 284.6 eV. The deconvolution of the high-resolution spectra was performed using the XPSPEAK41 program, in which an adjustment of the peaks was considered using peak fitting with Gaussian-Lorentzian peak shape and Shirley type background subtraction. The surface element compostion of sample were caculated based on the peak areas from high-resolution XPS spectra and the relative sensitivity factors. The relative sensitivity factors were selected from the Scofield element library in CasaXPS.

2. SERS measurement

Both the normal Raman spectrum and SERS spectra were collected using an HR 800 Raman spectroscope (J Y, France) equipped with a synapse CCD detector and a confocal

Olympus microscope. The 600 g/mm gratings and a 633 nm He-Ne laser with a power of about 20 mW were used in the spectrograph. All the spectra were obtained using LMPlanFl 50×, 0.5 numerical-aperture objective Lens (lens with the long focal length) with an accumulation time of 1 s.

The SERS measurements were carried out as follows. The Ge wafer grafted with Ag-Pt NPs was placed in a quartz cell with a quartz window. The cell was filled up with 100 μL 1×10^{-8} M R6G aqueous solution. During the SERS detection, the laser was focused on the Ag-Pt NPs through the quartz window.

3. The high magnification SEM images of Ag-Pt nanoparticles grafted on the Ge wafer

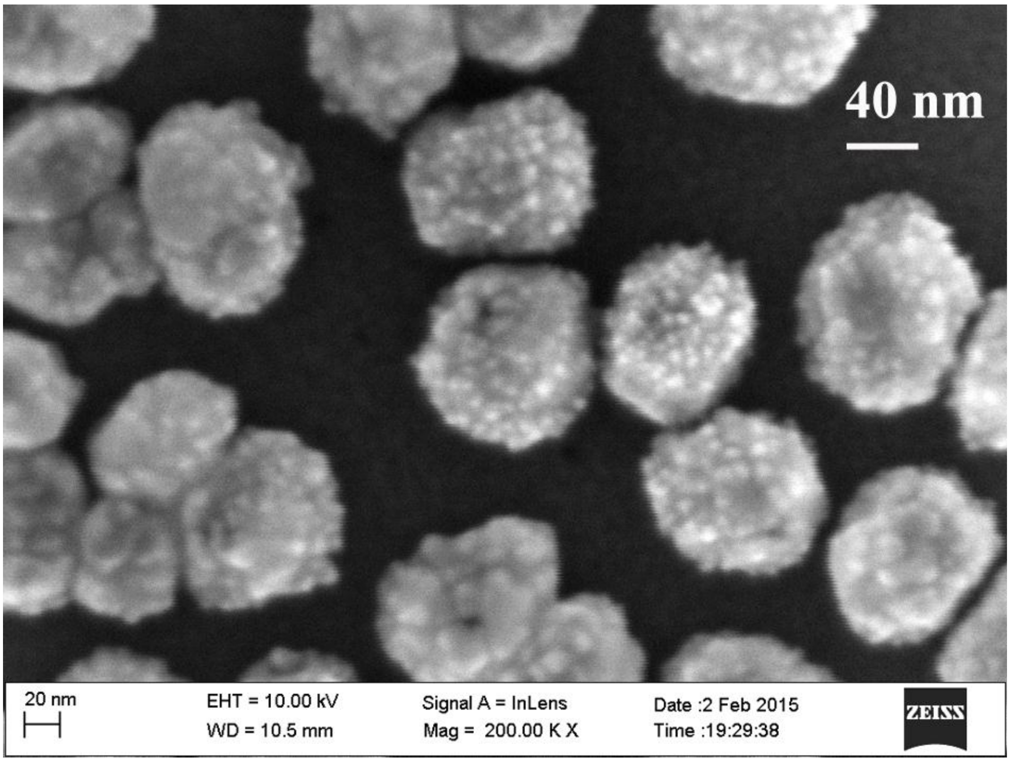

Figure S1. The high magnification SEM images of Ag-Pt nanoparticles grafted on the Ge wafer.

4. The EDS spectra of the Ag nanoparticle in the Ag-Pt/ Ge substrate

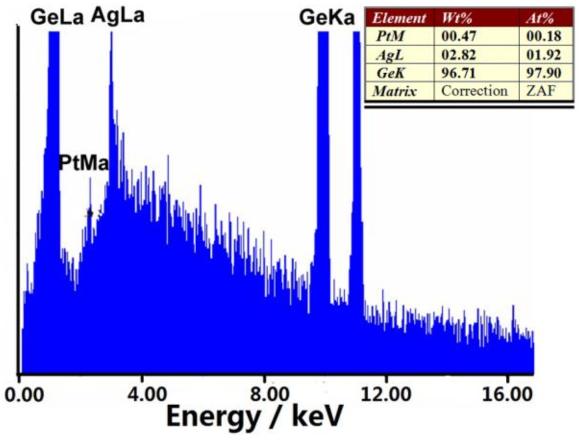

Figure S2. The EDS spectra of the the Ge wafer grown with Ag-Pt nanoparticles.

5. The normal Raman spectrum of 0.01 M R6G methanol solution

Fig. S3 shows the characteristics of the Raman spectra of 0.01 M R6G methanol solution. The C-C stretching vibration bending mode is 1181 cm^{-1}. The bands at 1366, 1511, 1580, and 1654 cm^{-1} are assigned to the aromatic C-C stretching modes.

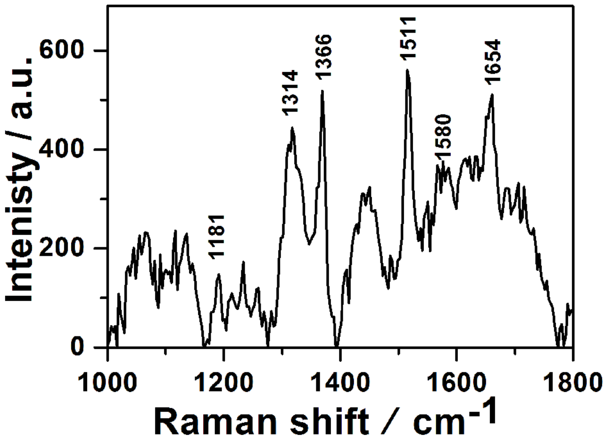

Figure S3. The normal Raman spectrum of 0.01 M R6G methanol solution.

6. The intensities of the major peaks in the 100 SERS spectra of R6G solution (1 × 10^{-8} M).

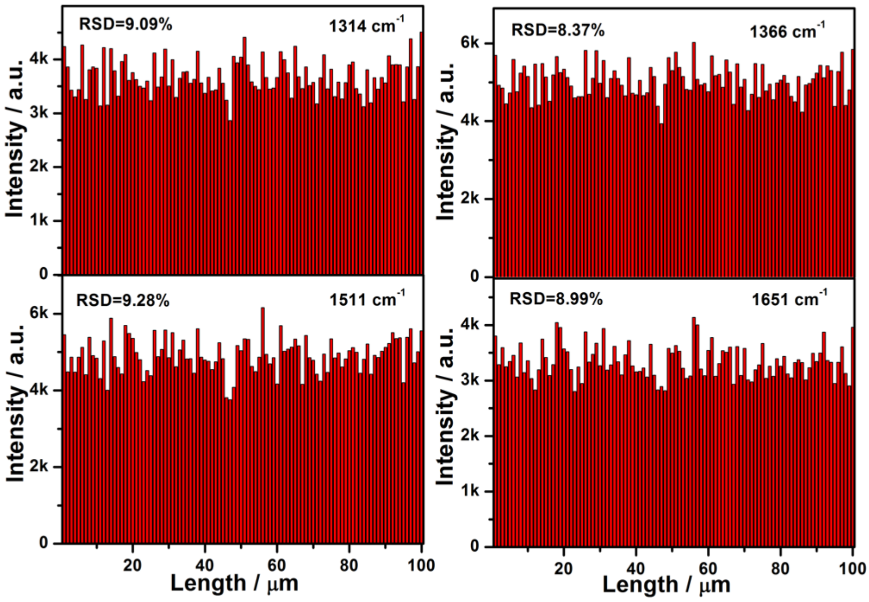

Figure S4. The intensities of the major peaks in the 100 SERS spectra of R6G solution (1 × 10^{-8} M).

7. Histograms of normalized Raman intensities of R6G (1 × 10^{-8} M) on Ag-Pt NPs/Ge SERS substrate

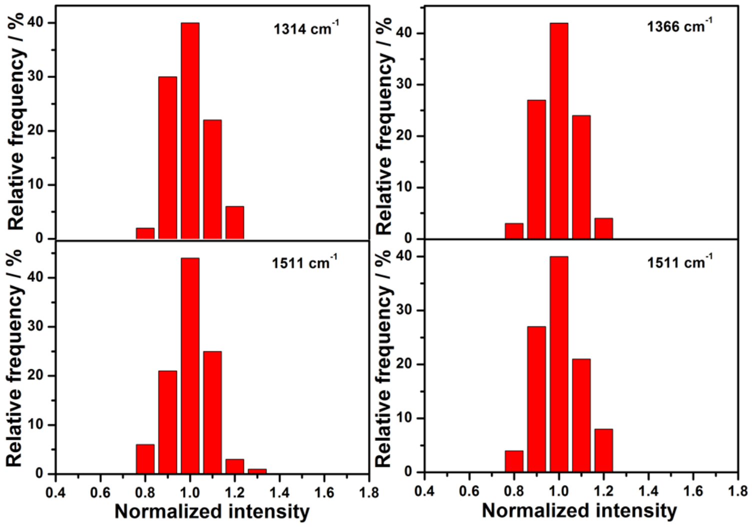

Figure S5. Histograms of normalized Raman intensities of R6G (1 × 10^{-8} M) on Ag-Pt NPs/Ge SERS substrate.

8. EF calculation of the as-prepared Ag-Pt NPs/Ge substrate:

The average SERS EF was calculated according to the formula:

$$\mathrm{EF}=\frac{I_{\mathrm{SERS}}N_0}{I_0 N_{\mathrm{SERS}}}$$

where I_0 and I_{SERS} are the peak intensity of the Raman measurement with 0.01 M R6G solution and SERS measurement with 1×10^{-8} M R6G solution, respectively; N_0 and N_{SERS} are the number of R6G molecules in the scattering volume for the Raman measurement and SERS measurement, respectively.

$$N_0 = n_0 N_A = C_0 V_0 N_A;$$

$$N_{SERS} = n_{SERS} N_A = C_{SERS} V_{SERS} N_A;$$

So, $$EF = \frac{I_{SERS}N_0}{I_0 N_{SERS}} = \frac{I_{SERS}C_0 V_0 N_A}{I_0 C_{SERS} V_{SERS} N_A} = \frac{I_{SERS}C_0}{I_0 C_{SERS}} = \frac{4922\times1\times10^{-2}}{540\times1\times10^{-8}} = 9.1\times10^{6};$$

where n_0 and n_{SERS} are the amount substance of R6G molecules in the scattering volume; V_0 and V_{SERS} are the scattering volume ($V_0 = V_{\mathrm{SERS}}$); C_0 and C_{SERS} are the concentration of R6G solution. The subscripts 0 and SERS represent Raman measurement and SERS measurement, respectively. A is the area of laser spot; h is the laser spot depth of focus; N_{A} is Avogadro constant.

10. The intensities of the major peaks in the 100 SERS spectra of 1 × 10^{-7} M CV solution

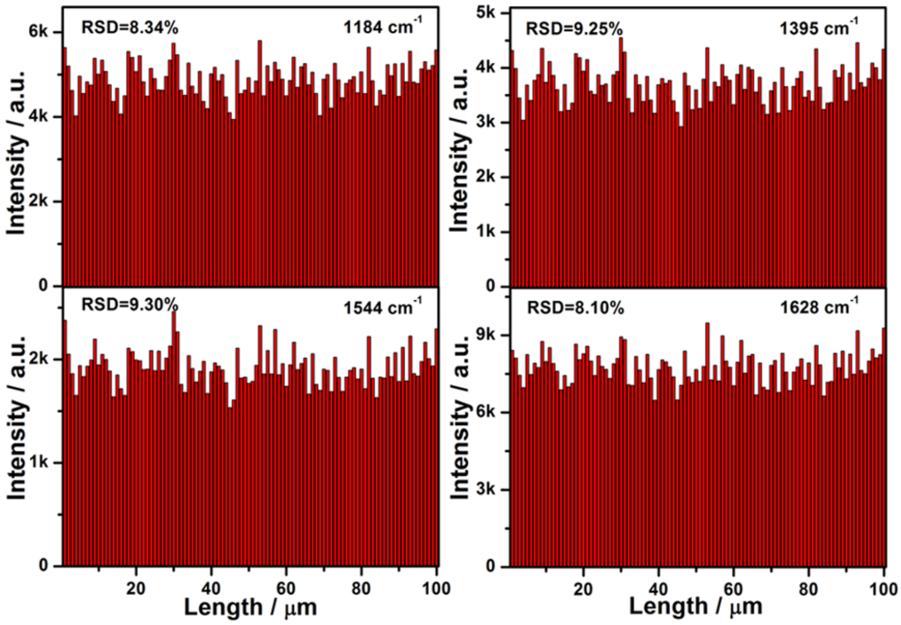

Figure S6. The intensities of the major peaks in the 100 SERS spectra of 1 × 10^{-7} M CV solution.

10. Histograms of normalized Raman intensities of CV (1 × 10^{-7} M) on Ag-Pt NPs/Ge SERS substrate

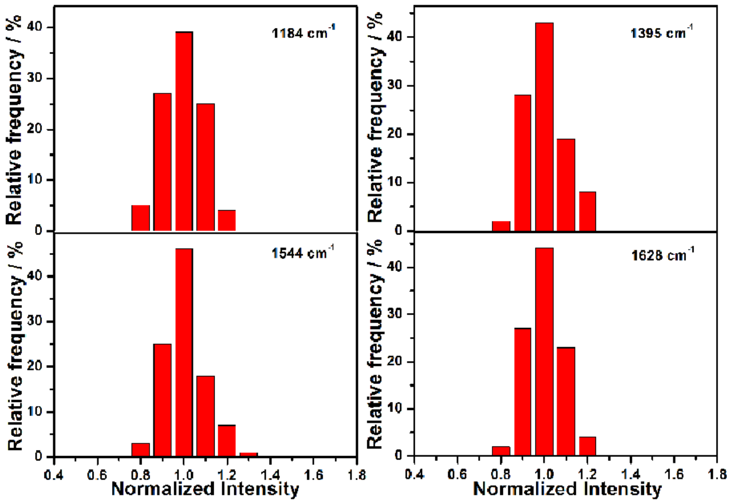

Figure S7. Histograms of normalized Raman intensities of CV (1 × 10^{-7} M) on Ag-Pt NPs/Ge SERS substrate.

11. **Histograms of normalized Raman intensities of R6G (1 × 10-8 M) on Ag-Pt NPs/Ge SERS substrate after a one-year storage in atmospheres at room temperature.**

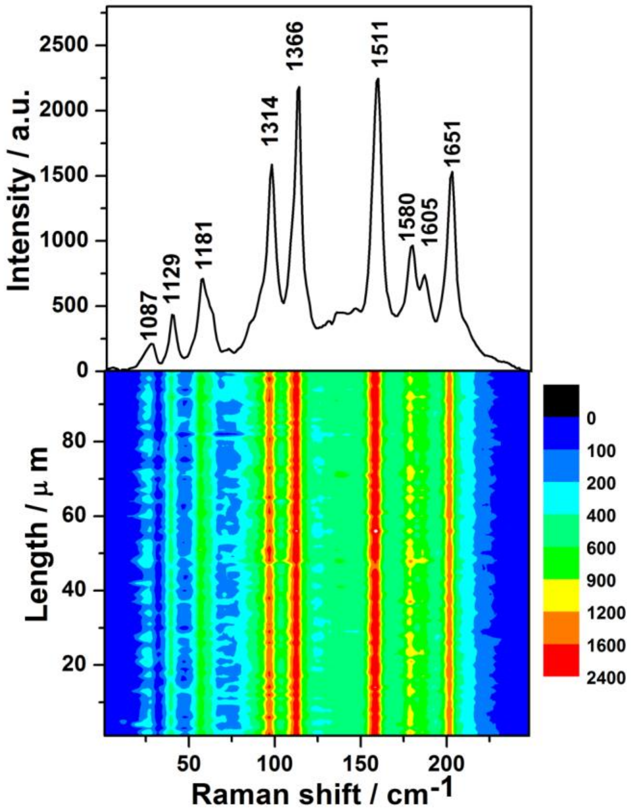

Figure S8. (Upper) the SERS spectrum of 1 × 10^{-8} M R6G solution on Ag-Pt NPs/Ge SERS substrate after a one-year storage in atmospheres at room temperature. (Lower) the SERS contour from line mapping of 100 spots.
